# Heterozygous Deletion of Epilepsy Gene *KCNQ2* Has Negligible Effects on Learning and Memory

**DOI:** 10.3389/fnbeh.2022.930216

**Published:** 2022-07-19

**Authors:** Gregory C. Tracy, Angelina R. Wilton, Justin S. Rhodes, Hee Jung Chung

**Affiliations:** ^1^Department of Molecular and Integrative Physiology, University of Illinois at Urbana-Champaign, Urbana, IL, United States; ^2^Beckman Institute for Advanced Science and Technology, University of Illinois at Urbana-Champaign, Urbana, IL, United States; ^3^Department of Psychology, University of Illinois at Urbana-Champaign, Urbana, IL, United States; ^4^Neuroscience Program, University of Illinois at Urbana-Champaign, Urbana, IL, United States

**Keywords:** *KCNQ2*, K_v_7 channel, learning, memory, nociception

## Abstract

Neuronal K_v_7/Potassium Voltage-Gated Channel Subfamily Q (KCNQ) potassium channels underlie M-current that potently suppresses repetitive and burst firing of action potentials (APs). They are mostly heterotetramers of K_v_7.2 and K_v_7.3 subunits in the hippocampus and cortex, the brain regions important for cognition and behavior. Underscoring their critical roles in inhibiting neuronal excitability, autosomal dominantly inherited mutations in Potassium Voltage-Gated Channel Subfamily Q Member 2 (*KCNQ2*) and Potassium Voltage-Gated Channel Subfamily Q Member 3 (*KCNQ3*) genes are associated with benign familial neonatal epilepsy (BFNE) in which most seizures spontaneously remit within months without cognitive deficits. *De novo* mutations in *KCNQ2* also cause epileptic encephalopathy (EE), which is characterized by persistent seizures that are often drug refractory, neurodevelopmental delay, and intellectual disability. Heterozygous expression of EE variants of *KCNQ2* is recently shown to induce spontaneous seizures and cognitive deficit in mice, although it is unclear whether this cognitive deficit is caused directly by K_v_7 disruption or by persistent seizures in the developing brain as a consequence of K_v_7 disruption. In this study, we examined the role of K_v_7 channels in learning and memory by behavioral phenotyping of the *KCNQ*2^+/−^ mice, which lack a single copy of *KCNQ2* but dos not display spontaneous seizures. We found that both *KCNQ*2^+/−^ and wild-type (WT) mice showed comparable nociception in the tail-flick assay and fear-induced learning and memory during a passive inhibitory avoidance (IA) test and contextual fear conditioning (CFC). Both genotypes displayed similar object location and recognition memory. These findings together provide evidence that heterozygous loss of *KCNQ2* has minimal effects on learning or memory in mice in the absence of spontaneous seizures.

## Introduction

Voltage-gated potassium (K^+^) channel subfamily Q (K_v_7/Potassium Voltage-Gated Channel Subfamily Q [KCNQ]) is a critical regulator of neuronal excitability (Greene and Hoshi, 2017; Baculis et al., 2020). In the central nervous system, K_v_7 channels are mostly heterotetramers of K_v_7.2 and K_v_7.3 subunits and to a lesser extent, heterotetrameric K_v_7.3 and K_v_7.5 channels and homomeric K_v_7.2 channels (Baculis et al., 2020). K_v_7.2 and K_v_7.3 show overlapping expression in the hippocampus and cortex, the brain regions critical for cognition and behavior (Cooper et al., 2001; Pan et al., 2006), and are highly concentrated at the axonal plasma membrane that include the initial segment when compared to the dendritic plasma membrane (Chung et al., 2006; Pan et al., 2006). Upon membrane depolarization, K_v_7 channels mediate slowly activating and non-inactivating outward K^+^ current called M-current (*I*_M_) (Brown and Passmore, 2009) that potently suppresses action potential (AP) firing rate and burst firing, hyperpolarizes resting membrane potential, and regulates spike threshold and after hyperpolarization (Aiken et al., 1995; Gu et al., 2005; Shah et al., 2008; Greene and Hoshi, 2017; Baculis et al., 2020). Additionally, K_v_7 channels in hippocampal pyramidal neurons produce intrinsic theta resonance called M-resonance at depolarized subthreshold potentials (Peters et al., 2005).

Underscoring the critical roles of K_v_7 channels in inhibiting neuronal excitability (Greene and Hoshi, 2017; Baculis et al., 2020), the agonist retigabine reduces seizures in animal models and humans (Miceli et al., 2008), whereas dominant mutations in either Potassium Voltage-Gated Channel Subfamily Q Member 2 (*KCNQ2*) or Potassium Voltage-Gated Channel Subfamily Q Member 3 (*KCNQ3*) genes cause neonatal epilepsy that includes benign familial neonatal epilepsy (BFNE) and epileptic encephalopathy (EE) (www.rikee.org, www.ncbi.nlm.nih.gov/clinvar/). In most patients with BFNE, neonatal seizures fully abate within weeks to months after birth (Miceli et al., 2011; Soldovieri et al., 2011). In contrast, patients with EE display early-onset intractable seizures, which are often drug resistant (Weckhuysen et al., 2012; Nappi et al., 2020). Most BFNE and EE mutations impair voltage-dependent activation, phosphatidylinositol bisphosphate (PIP_2_) sensitivity, and/or axonal enrichment of K_v_7 channels (Miceli et al., 2011; Soldovieri et al., 2011; Weckhuysen et al., 2012; Cavaretta et al., 2014; Kim et al., 2018; Nappi et al., 2020; Zhang et al., 2020). Furthermore, heterozygous knock-in mice for BFNE mutants K_v_7.2^Y284C^, K_v_7.2^A306T^, or K_v_7.3^G311V^ show heightened seizure susceptibility (Singh et al., 1998, 2008), whereas heterozygous expression of the EE variant K_v_7.2^T274M^ or K_v_7.2^M547V^ induces spontaneous seizures and early mortality in mice (Milh et al., 2020; Kim et al., 2021), suggesting that K_v_7 dysregulation contributes to BFNE and EE.

In addition to seizures, patients with EE develop neurodevelopmental delay and intellectual disability (Zhang et al., 2020) and *de novo* dominant mutations in *KCNQ2* and *KCNQ3* genes have recently been associated with neurodevelopmental delay without seizures (Coe et al., 2019), suggesting the possible role of K_v_7 channels in learning and memory (Baculis et al., 2020). Indeed, K_v_7 channel antagonists, linopirdine and XE991, enhance fear-motivated avoidance learning and object recognition task performance in rodents in the mouse model of dementia (Cook et al., 1990; Fontan-Lozano et al., 2011). Stimulation of Gq-coupled muscarinic acetylcholine receptors (mAChRs) and subsequent inhibition of *I*_M_ (Brown and Passmore, 2009) in the prefrontal cortex (PFC) prevents the decline in working memory induced by cholinergic depletion in aging primates (Galvin et al., 2020). In contrast to these beneficial effects of acute pharmacological inhibition of K_v_7 channels on cognition, genetic knock-down or ablation of a single Drosophila Potassium Voltage-Gated Channel Subfamily Q Member 1 (*dKCNQ*) gene in *Drosophila* is shown to induce ethanol hyperexcitability (Cavaliere et al., 2012) and impair both short-term memory and long-term memory, respectively (Cavaliere et al., 2013). In mice, genetic suppression of *I*_M_ by overexpression of the dominant negative mutant K_v_7.2^G279S^ or EE mutant K_v_7.2^T274M^ or K_v_7.2^M547V^ in the developing brain induces spontaneous seizures and impairs learning and memory (Peters et al., 2005; Milh et al., 2020; Kim et al., 2021). However, it is difficult to tease out whether cognitive deficits in the genetic mouse models arise directly from the reduction of *I*_M_ or from indirect consequences of persistent seizures induced by *I*_M_ suppression.

Here, we investigated the role of K_v_7.2-containing channels in learning and memory by behavioral phenotyping of the *KCNQ*2^+/−^ mice that were heterozygous null for *KCNQ2* but show a normal level of *KCNQ3* transcript (Tzingounis and Nicoll, 2008). Consistently, *KCNQ*2^+/−^ mice display reduced K_v_7.2 but not K_v_7.3 expression in their hippocampi when compared to the wild-type (WT) mice (Kim et al., 2019). Although *I*_M_ in *KCNQ*2^+/−^ mice as compared to WT mice has not been reported, the *KCNQ*2^+/−^ dentate granule cells display a 50% decrease in the amplitudes of medium and slow after hyperpolarization currents (Tzingounis and Nicoll, 2008), suggesting the contribution of K_v_7.2-containing channels. *KCNQ*2^+/−^ mice were chosen because they are viable and do not show spontaneous seizures (Watanabe et al., 2000; Tzingounis and Nicoll, 2008) in sharp contrast to homozygous *KCNQ2* knock-out mice that are perinatal lethal (Watanabe et al., 2000; Tzingounis and Nicoll, 2008) and conditional homozygous forebrain knock-out mice of *KCNQ2*, which display spontaneous seizures and early mortality by weaning age (Soh et al., 2014). In addition, *KCNQ*2^+/−^ mice and WT *KCNQ*2^+/+^ mice show comparable levels of locomotor activity and motor coordination (Kim et al., 2019), making *KCNQ*2^+/−^ mice a suitable model to study the effect of K_v_7.2 haploinsufficiency on learning and memory in the absence of spontaneous seizures. In this study, we discovered that fear-motivated learning and memory, spatial memory, and object recognition memory were unaffected by the heterozygous loss of *KCNQ2*. Interestingly, *KCNQ*2^+/−^ mice display a longer total time to reach the criterion than the WT mice during the acquisition phase of the inhibitory avoidance (IA) task, suggesting a possible specific deficit in decision-making and/or fear perception.

## Materials and Methods

### Experimental Animals

All animal procedures were approved by the Institutional Animal Care and Use Committee of the University of Illinois at Urbana Champaign. *KCNQ*2^+/−^ mice on the C57BL/6J background have been obtained from the Jackson Laboratory [*Kcnq*2^tm1Dgen^/*Kcnq*2^+^, Stock Number: 005830 (Tzingounis and Nicoll, 2008)]. *KCNQ*2^+/−^ mice were bred against C57BL/6J mice and housed on a normal 12:12 light:dark cycle (lights on at 6 a.m. and lights off at 6 p.m.) with food and water available *ad libitum*. At weaning, the littermates of the same sex were group-housed with up to 5 mice per cage and were genotyped as described (Kim et al., 2019).

### Behavior Studies

A total of 66 male mice were used (*KCNQ*2^+/+^ *n* = 37; *KCNQ*2^+/−^ *n* = 29) and a total of 78 female mice were used (*KCNQ*2^+/+^ = 36; *KCNQ*2^+/−^ *n* = 42) for behavior tests at 4–6 months of age. Experimenters were blind to the mouse genotype. The motor coordination of *KCNQ*2^+/−^ mice has been previously reported to be similar to their WT littermates (Kim et al., 2019). Because *KCNQ*2^+/−^ mice display hyperactivity in the light but not in dark phase (Kim et al., 2019), both genotypes were tested for the passive IA test, contextual fear conditioning (CFC), object location task (OLT), and novel object recognition task (NORT) during the dark phase in a separate “behavior” room from the rest of the colony. Specifically, these tasks were performed 2 h after the start of the dark phase. To eliminate possible pain-induced effects on future behaviors, OLT and NORT were followed by passive IA a minimum of 10 days later, and separate cohorts were used for CFC and tail-flick assay. Furthermore, we used both male and female mice since sex differences in social dominance and compulsive behavior were previously reported in *KCNQ2*^+/−^ mice (Kim et al., 2019).

Passive IA, OLT, and NORT were performed in a behavior room maintained on a “reverse” light:dark cycle (lights off at 10 a.m. and lights on at 10 p.m.) after the mice were single housed and habituated to both this cycle and handling for 2 weeks. Due to a conflict with the availability of the behavior room maintained on a reverse light:dark schedule, the mice for CFC were subjected to this task during the dark phase in a different behavior room maintained in a normal 12:12 light:dark cycle after habituation to the handling for 2 weeks. Compared to the dark phase, mice display higher pain sensitivity in the light phase (Kavaliers and Hirst, 1983). Therefore, the tail-flick assay was performed on both genotypes in the light phase, which may better reveal the genotype-specific difference in nociception. Hyperactivity of *KCNQ*2^+/−^ mice in the light phase as compared to the WT mice (Kim et al., 2019) is expected to have a negligible effect on the thermal nociception examined by the tail-flick assay, which does not require the locomotion of mice. All behavior apparatus was cleaned with 70% ethanol between each mouse.

The passive IA was performed in a dedicated behavior room as described (Hamilton et al., 2017). On Day 1 (training), the mouse was placed in a two-chamber GEMINI Avoidance System (San Diego Instruments) and the start button was immediately pressed to turn on the LED light in the chamber containing the mouse and simultaneously raise the gate separating the two chambers. When the mouse crossed into the dark chamber, the gate was closed and the mouse received a mild foot shock (0.5 mA) for 4 s (s). After 10 s, the LED house light was turned back on in the chamber containing the mouse and the gate was opened, allowing the mouse to cross to the dark chamber. This procedure was repeated until the mouse reached the criterion by remaining in the lit chamber for 120 s or until 50 attempts had been made without meeting the criterion. The latency to cross per trial and the number of crosses to reach the criterion during the training period were recorded. At 24 and 48 h after training, the mouse was placed again in the avoidance system without foot shocks, and the latency to cross to the dark chamber was recorded up to 300 s. If the mouse had not crossed into the dark chamber by 300 s, the trial was marked as a “no cross” and latency was recorded as 300 s.

The CFC task was performed as described (Kohman et al., 2012) in the chamber containing a metal grate that spanned the bottom of the box and administered a foot shock. On each day of experimentation, the mice were first separated into a single housing for 4–5 h in a separate behavior room and then tested starting at 2 h into the dark phase under video recording. On Day 1, the mouse was placed in the chamber for habituation for 180 s. At 24 h after habituation on Day 2 (training), the mouse was placed in the same chamber for 180 s during which a mild foot shock (0.5 mA, 2 s) was delivered at 120 and 150 s. At 24 h after training on Day 3 (testing), the mouse was placed in the same chamber for 180 s without foot shocks. The total freezing time was recorded on each day. Freezing was defined as a total lack of movement outside of breathing. At the end of experiments in each day, the mice were returned to their original group housing. The percentage (%) of total time spent freezing was calculated as $100\left(\frac{freezing\;time\;\left(s\right)}{180\;s}\;\right)$.

The OLT and NORT were performed with video recording as described (Denninger et al., 2018). On Day 1, the mouse was placed in a designated release corner of the empty test chamber and allowed to explore for three separate 6-min intervals. At 24 h after habituation on Day 2, the mouse was placed back into the chamber and allowed to explore the objects for 10 min in 3 different test phases. In phase 1 (training), the chamber contained two distinct objects secured at 6 × 6 cm^2^ from its two corners. In phase 2 (OLT), one of the objects was moved to a new location at the opposite corner. In phase 3 (NORT), the object that was previously moved in OLT was replaced with a novel object. After each phase, the mouse was placed in their holding cage for 20 min. TopScan (CleverSys) was used to track the movement of a mouse in the chamber and record the duration of its investigation of an object, which is defined as when its head was oriented toward the object within 1 cm or when its nose was touching the object. The discrimination index (DI) was calculated as $100\left(\frac{time\;sniffing\;novel\;object\;or\;location-time\;sniffing\;familiar\;object\;or\;location}{time\;sniffing\;novel+time\;sniffing\;familiar\;object\;or\;location}\right)$. The mice that have DI > +0.2 or < **–**0.2 during training were considered to have a significant location and/or object bias during training and were excluded from statistical analysis as previously described (Vogel-Ciernia and Wood, 2014).

The tail-flick assay was performed as described (Schildhaus et al., 2014). Two 500 ml beakers were filled with 450 ml of distilled water and placed on stir plates with induction heaters to maintain even water temperatures of 36 and 51°C. During the test, the stir plates were turned off and the tail of the mouse was lowered 3 cm into the 36°C bath for 30 s or until it started to flick rapidly. The latency until the tail-flick was recorded. The tail was dried and returned to room temperature. The same procedure was then repeated for the 51°C bath.

### Statistical Analysis

All analyses are reported as mean ± SEM. The n values indicate the number of mice. Origin Pro 9.5 (Origin Lab) was used to perform statistical analyses. When the data were separated by sex from passive IA and CFC, tail-flick tests (Supplementary Figures S1, S2) were analyzed using the 2-way ANOVA with genotype as one factor and sex as the other, there were no significant effects of sex and no interactions between sex and genotype (Supplementary Table S1). Therefore, the data for male mice and female mice were combined and analyzed by a two-tailed student's *t*-test for comparing 2 groups and a *post-hoc* Tukey test for comparing >2 groups. *A priori* value (*p*) < 0.05 was used to establish statistical significance.

## Results

### Heterozygous Loss of *KCNQ2* Has Minimal Effects on IA and Does Not Affect Contextual Fear-Induced Learning or Memory

**Figure 1:**
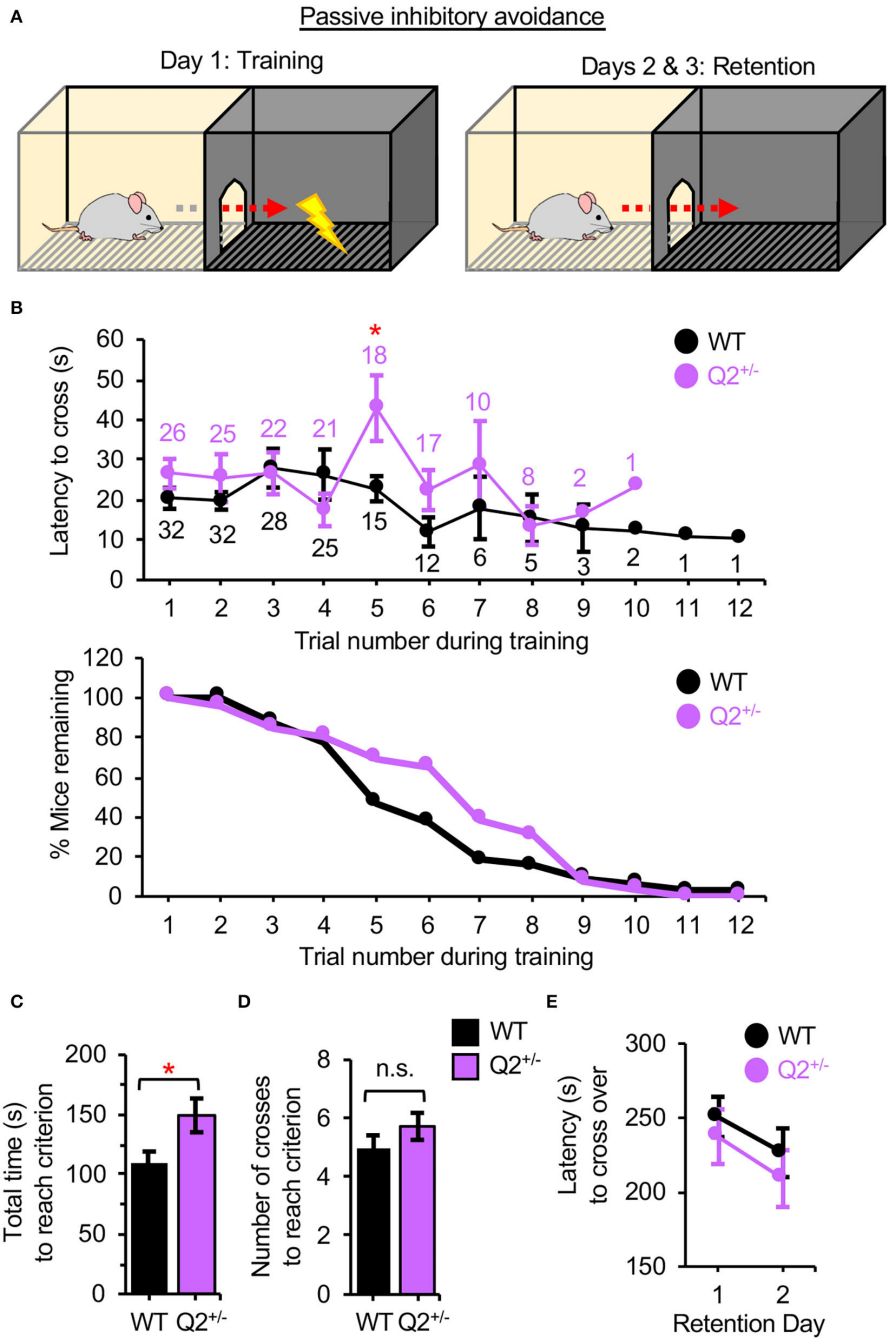
Heterozygous loss of Potassium Voltage-Gated Channel Subfamily Q Member 2 (*KCNQ2*) increases the total time to reach criterion during training on an inhibitory avoidance (IA) task but does not affect memory in mice. *KCNQ2*^+/+^ (wild-type; WT) mice and *KCNQ2*^+/−^ (Q2^+/–^) mice at age 4–6 months were subjected to passive IA test. **(A)** The design of the passive IA test. On the training day, the criterion for fear-motivated learning is established when the mouse remains in the lit chamber for 120 s rather than entering the dark chamber where it receives a foot shock (0.5 mA, 4 s). At 1 and 2 days after training (retention days), the trained mouse is placed in the lit chamber and fear-induced memory is tested by recording the latency to cross into the dark chamber for a maximum of 300 s. **(B)** The latency to cross into the dark chamber in each trial during training (top graph). The number of WT mice (black) and that of Q2^+/−^ mice (purple) that remained in each trial is also shown. The percentage (%) of mice remaining in each trial during training (bottom graph). **(C)** The total time to reach the criterion during the training was calculated by adding the latency per trial except for the final 120 s when the mouse remained in the lit chamber without crossing. **(D)** The number of crosses during the training. **(E)** The latency to cross into the dark chamber during retention days. Number of mice used: WT (*n* = 31 that includes 14 male mice and 17 female mice), Q2^+/−^ (*n* = 26 that includes 11 male mice and 15 female mice). Data represent the mean ± SEM. Two-tailed student's *t*-test results are shown (*p < 0.05). The individual data points are shown in Supplementary Figure S1.

To test the role of K_v_7.2-containing channels in fear-motivated learning and memory in mice, adult *KCNQ*2^+/−^ mice, and their WT littermates (*KCNQ2*^+/+^) at 4–6 months of age were subjected to the passive IA and CFC. These tasks are dependent on the hippocampus, amygdala, and PFC (Kohman et al., 2012; Hamilton et al., 2017). In the passive IA, which exploits a rodent's natural preference for the dark environment (Hamilton et al., 2017), the criterion for fear-motivated learning is established during training when the mouse remains in the lit chamber for 120 s rather than entering the dark chamber where it receives a foot shock (Figure 1A). We found that *KCNQ*2^+/−^ mice took a longer time to cross in the fifth trial and displayed a longer total time to reach the criterion during training as compared to *KCNQ2*^+/+^ mice (Figures 1B,C, Supplementary Figure S1). These results together suggest that *KCNQ*2^+/−^ mice were more hesitant to cross into the dark chamber where they previously received a shock. However, this did not translate into better learning of the task because both genotypes displayed a similar number of crosses to reach the criterion (Figure 1D, Supplementary Figure S1). Both genotypes also showed comparable latency to enter the dark chamber at 1–2 days after training (Figure 1E, Supplementary Figure S1), indicating that fear-induced memory on the IA task was unaffected by heterozygous loss of *KCNQ2*.

**Figure 2:**
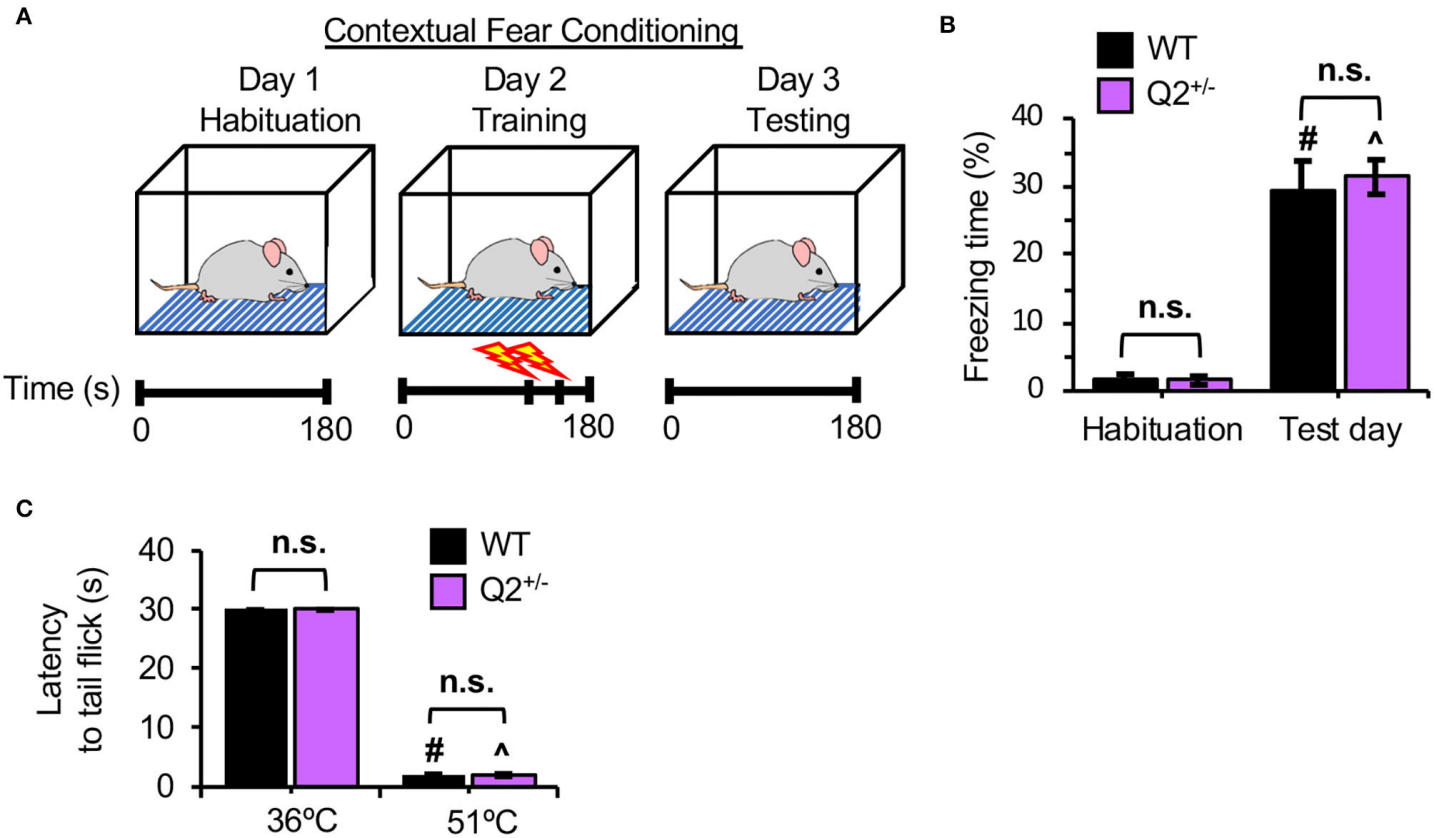
Heterozygous loss of Potassium Voltage-Gated Channel Subfamily Q Member 2 (*KCNQ2*) in mice does not affect fear memory retention and thermal pain threshold. Separate cohorts of *KCNQ2*^+/+^ (wild-type; WT) mice and *KCNQ2*^+/−^ (Q2^+/−^) mice at age 4–6 months were subjected to contextual fear conditioning (CFC) test and tail-flick assay. **(A)** The design of the CFC test. A mouse is first habituated in a CFC chamber for 180 s. Next day (training day), the mouse is placed in the chamber for 180 s and receives a mild foot shock (0.5 mA, 2 s) at 120 s and 150. On 1 day after foot shocks (Test day), a mouse is returned to the chamber for 180 s. Freezing time is recorded on habituation and retention days. **(B)** Freezing time is the percentage (%) of total time spent in the chamber. The number of mice used: WT (*n* = 20 that includes 10 male mice and 10 female mice) Q2^+/−^ (*n* = 20 that includes 10 male mice and 10 female mice). *Post-hoc* Tukey test results are shown for the habituation day vs. the test day in WT (^#^*p* < 0.001) and in Q2^+/−^ (^∧^*p* < 0.001). **(C)** The average latency to tail-flick. In tail-flick assay, a mouse is restrained in a 50 ml conical tube and the tail is lower into a 36°C water bath and the latency to tail-flick is recorded for 30 s. The procedure is repeated with a 51°C water bath. All mice reached 30 s latency at 36°C. The number of mice used: WT (*n* = 20 includes 10 male mice and 10 female mice), Q2^+/−^ (*n* = 20 includes 9 male mice and 11 female mice). Data represent the mean ± SEM. *Post-hoc* Tukey test results are shown for 36°C vs. 51°C in WT (^#^*p* < 0.001) and in Q2^+/−^ (^∧^*p* < 0.001). No significant difference between genotypes was found (n.s.). The individual data points are shown in Supplementary Figure S2.

To further investigate the role of K_v_7.2-containing channels in fear-induced memory, CFC was performed (Kohman et al., 2012). This task tests the ability of a mouse to remember and associate the CFC chamber (context) with the foot shocks (aversive stimuli) (Figure 2A). During habituation in the CFC chamber without foot shocks, both *KCNQ2*^+/+^ and *KCNQ2*^+/−^ mice displayed minimal freezing (Figure 2B, Supplementary Figure S2A). At 1-day post-foot shocks, the freezing response duration was significantly increased in both genotypes to a similar extent (Figure 2B, Supplementary Figure S2B), indicating that heterozygous loss of *KCNQ2* does not affect contextual fear memory.

### Heterozygous Loss of *KCNQ2* Does Not Affect Thermal Pain Sensitivity in Mice

A subtle difference in the total time to reach the criterion in passive IA could be caused by the difference in nociception between *KCNQ2*^+/−^ and the WT mice, since functional K_v_7 channels exist in dorsal root ganglia neurons (Rose et al., 2011). To test this, we performed the tail-flick assay (Schildhaus et al., 2014) in which the latency to the tail-flick was measured upon incubating the mouse tail in the 51°C water bath as compared to the control in 36°C water bath. We found that both *KCNQ*2^+/+^ and *KCNQ*2^+/−^ mice displayed similar latency to the tail-flick in a hot water bath (Figure 2C, Supplementary Figure S2C), indicating no significant genotype difference in thermal pain tolerance.

### Heterozygous Loss of *KCNQ2* Does Not Affect Object Location and Recognition Memory in Male Mice

**Figure 3:**
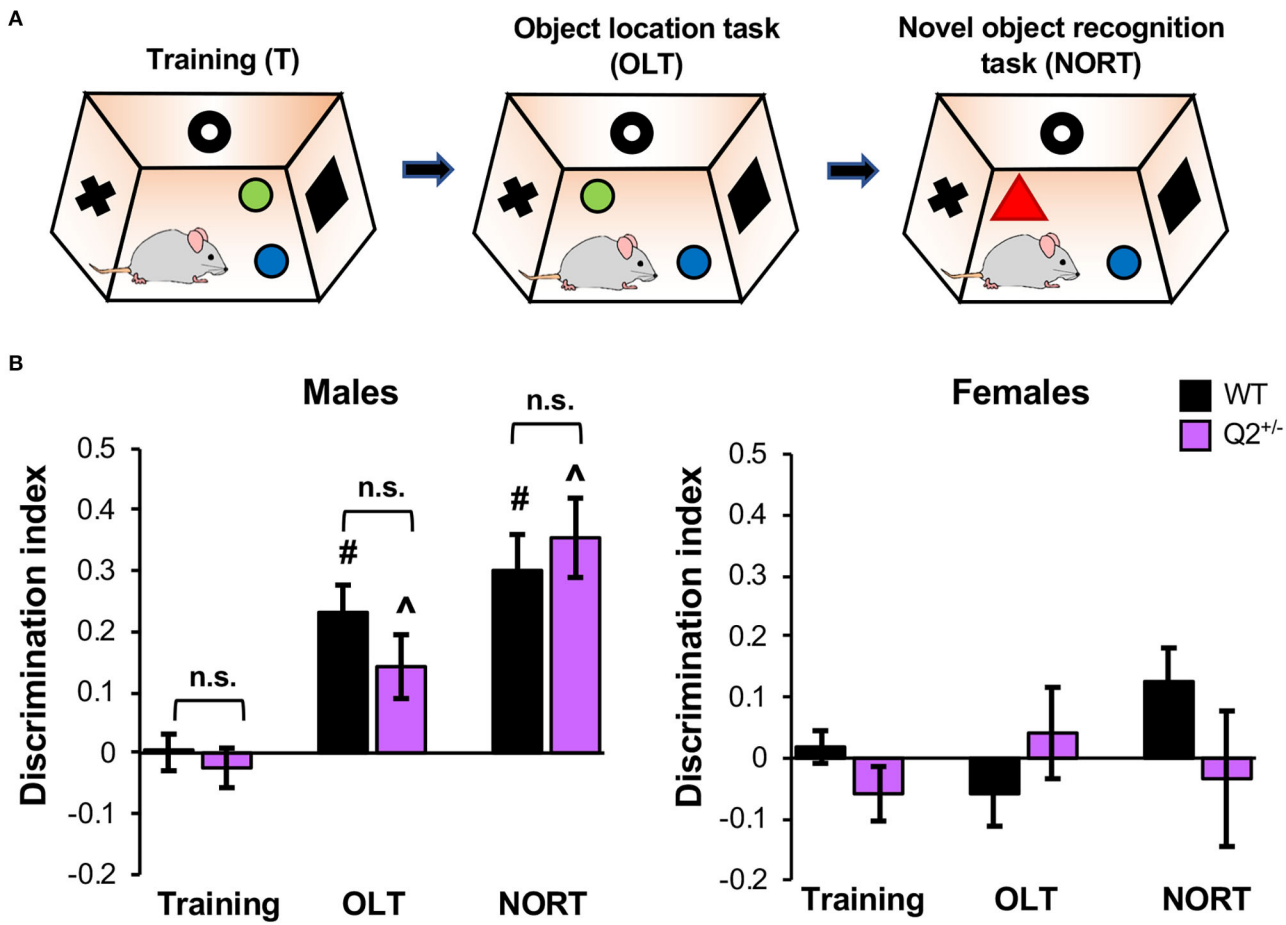
Heterozygous loss of Potassium Voltage-Gated Channel Subfamily Q Member 2 (*KCNQ2*) does not affect object location and recognition memory in male mice. *KCNQ2*^+/+^ (wild-type; WT) mice and *KCNQ2*^+/−^ (Q2^+/−^) mice at age 4–6 months were subjected to the object location task (OLT) and novel object recognition task (NORT). **(A)** The design of the OLT and NORT. **(B)** Discrimination index (DI) of male mice during OLT and NORT. During OLT and NORT, positive DI indicates the preference for the novel object or location, negative score equals preference for a familiar object or location, and zero score indicates no preference. The number of mice used: male mice (WT = 14, Q2^+/−^ = 8), female mice (WT = 12, Q2^+/−^ = 7). Data represent the mean ± SEM. *Post-hoc* Tukey test results are shown for training vs. OLT or training vs. NORT for WT male mice (^#^*p* < 0.001) and for Q2^+/−^ male mice (^∧^*p* < 0.001). The individual data points are shown in Supplementary Figure S3.

To test if heterozygous loss of *KCNQ2* affects memory that is not induced by fear, we next performed the OLT, which evaluates hippocampus-dependent spatial memory, and the NORT, which tests non-spatial memory of object identity (Vogel-Ciernia and Wood, 2014; Denninger et al., 2018). In these tasks, a mouse is first habituated to an empty test chamber. The next day, the mouse explores two distinct objects in the same chamber for 10 min and then is removed for 20 min (training, Figure 3A). The mouse returns to the same chamber where one of the objects was moved and explores for 10 min (OLT, Figure 3A). After a 20-min break, the mouse returns to the same chamber where one of the objects is replaced with a novel object and explores for 10 min (NORT, Figure 3A). After removing mice that showed a significant bias for one object over the other during training as indicated by 0.2 < DI < −0.2 (see Supplementary Figure S3B for percentages of mice that met this criterion), the 2-way ANOVA for both OLT and NORT showed a significant effect of sex and interaction between genotype and sex (Supplementary Table S1). The criterion of 0.2 was chosen as the threshold for object bias during training based on Vogel-Ciernia and Wood (2014) since typical DIs for short- and long-term memory range from 0.25 to 0.45 (Vogel-Ciernia and Wood, 2014).

Inspection of the graphs showed that the male groups displayed significant object location and novel object recognition (Figure 3B). A small portion of male mice of both genotypes did not perform well as compared to the training phase (Supplementary Figure S3C). Since both genotypes display similar motor coordination and travel comparable distance in the open field arena in the dark phase (Kim et al., 2019) and had been habituated for 2 weeks to the handling and reverse light:dark cycle, we speculate that the unexpected environmental stress factors (the noise from the building or a cage change by an animal facility technician) might have contributed to this low performance of a small portion of male mice.

In addition, the female groups failed to show significant object location or novel object recognition as indicated by average DI not significantly different from zero (Figure 3B). Therefore, we concluded that female mice failed to display adequate performance on the task for analysis. Previous studies have shown that female mice trained outside the proestrus stage did not recognize the new object location 1 day after training whereas female mice trained in proestrus performed well in OLT similar to male mice (Gall et al., 2021). Although we did not monitor the estrous cycle in female mice, we speculate that their deficits in OLT and NORT might have resulted from different stages in their estrous cycle, which could have had varying effects on their exploratory behavior and/or spatial and object memory (Gall et al., 2021).

Because female mice failed to perform the OLT and NORT tasks, male mice were analyzed alone. Results of the *t*-test comparing DI for OLT and NORT in male mice showed no significant effect of genotype (Supplementary Table S2, Figure 3B). This result suggests that heterozygous loss of *KCNQ2* does not affect spatial memory and object recognition memory in male mice.

## Discussion

In this study, we performed behavioral phenotyping of the *KCNQ2*^+/−^ mice, which lack half of the *KCNQ2* transcript and display reduced K_v_7.2 expression but show no spontaneous seizures (Watanabe et al., 2000; Kim et al., 2019). Although *KCNQ*2^+/−^ mice have been previously shown to display enhanced exploratory and repetitive behaviors and reduced sociability in both sexes as compared to *KCNQ*2^+/+^ mice (Kim et al., 2019), this study provides the first evidence that heterozygous loss of *KCNQ2* does not affect fear-induced learning and memory on the IA task and CFC in both sexes (Figures 1, 2) nor object location or recognition memories in male mice (Figure 3).

Interestingly, although both genotypes displayed a similar number of crosses to reach the criterion (Figure 1D), we observed that *KCNQ2*^+/−^ mice took longer to reach the criterion during training especially in the fifth trial in the IA task than *KCNQ2*^+/+^ mice (Figures 1B,C), suggesting that *KCNQ2*^+/−^ mice tended to delay crossing to the dark chamber per trial compared to the WT mice. The interpretation of this difference is unclear. We speculate that *KCNQ2*^+/−^ mice tend to delay crossing to the dark chamber per trial as compared to the WT mice, reflecting ambivalence in the decision-making during the phase of the fear-motivated learning (Figure 1B).

However, it is unclear how heterozygous loss of K_v_7.2 could affect the decision-making during the phase of fear-motivated learning. Previous studies have shown that neonatal exposure to K_v_7 opener retigabine and linopirdine does not affect basal nociceptive sensitivity of rats in the tail-flick assay (Frankel et al., 2016) and upon a foot shock (Cook et al., 1990), respectively. Application of K_v_7 antagonist XE991 also does not increase thermal hyperalgesia in a rat model of neuropathic injury (Rose et al., 2011). Similar to these pharmacological studies, we found that *KCNQ2*^+/−^ mice displayed similar thermal nociception as the WT mice in tail-flick assay (Figure 2C). Thus, the effect of reduced *I*_M_ in this phase of decision-making could arise not from the difference in nociception. Rather, it could arise from hyperexcitability of the temporal lobe involved in passive IA because *KCNQ2*^+/−^ mice show a heightened seizure susceptibility against kainic acid (Kim et al., 2019), which induces status epilepticus arising from the temporal lobe (Levesque and Avoli, 2013). Alternatively, considering that K_v_7.2 and K_v_7.3 are present in medial PFC (Pan et al., 2006), which suppresses amygdala outputs (Quirk et al., 2003), the slightly lengthened decision-making time in *KCNQ*2^+/−^ mice could result from the increased activity of medial PFC by reduced K_v_7.2 expression, which in turn inhibits the amygdala-dependent perception of fear (Etkin and Wager, 2007).

Minimal disruption of memory in *KCNQ*2^+/−^ mice (Figures 1–3), which lacks spontaneous seizures (Watanabe et al., 2000; Kim et al., 2019), is in sharp contrast to cognitive deficits induced by genetic ablation of *I*_M_ in mice, which display spontaneous seizures (Peters et al., 2005; Milh et al., 2020; Kim et al., 2021). For example, dominant negative suppression of *I*_M_ by transgenic overexpression of the dominant negative mutant K_v_7.2^G279S^ or heterozygous expression of dominant negative EE mutants K_v_7.2^T274M^ and K_v_7.2^M547V^ in the developing brain induces spontaneous seizures, impaired hippocampus-dependent spatial memory, and object recognition memory (Peters et al., 2005; Milh et al., 2020; Kim et al., 2021). Considering that *KCNQ2* and *KCNQ3* expressions begin during embryonic development (Dirkx et al., 2020), we propose that K_v_7 channels contribute to normal brain development and a significant reduction in K_v_7 current early in the development may be necessary to disrupt proper circuit formation critical for cognition alone (Watanabe et al., 2000; Soh et al., 2014) or to induce spontaneous seizures, which could exacerbate this disruption.

In contrast to genetic models, pharmacological studies have revealed conflicting roles of K_v_7 channels in learning and memory. K_v_7 antagonist XE991 is shown to reduce the induction threshold of long-term potentiation (LTP) of excitatory synaptic strength at hippocampal CA1–CA3 synapses (Song et al., 2009; Fontan-Lozano et al., 2011), suggesting a facilitating role of K_v_7 inhibition in LTP, which mediates hippocampus-dependent learning and memory (Whitlock et al., 2006). Consistently, K_v_7 antagonists enhance fear memory and block memory impairments induced by hypoxia (Cook et al., 1990) and cholinergic depletion (Fontan-Lozano et al., 2011). In contrast, K_v_7 channel opener retigabine is shown to inhibit the stress-induced reduction in hippocampus-dependent spatial memory (Li et al., 2014). These studies suggest that memory can be enhanced by either acute inhibition or enhancement of *I*_M_ depending on the underlying circuitry and the pathological condition. Given that the tauopathy mouse model of dementia displays reduced frontotemporal expression of K_v_7 subunits (de Jong and Jepps, 2018), future studies shall further explore the precise role of *I*_M_ on age-related dementia and AD.

## Data Availability Statement

The original contributions presented in the study are included in the article/Supplementary Materials, further inquiries can be directed to the corresponding author. The data excel file is also available in figshare: https://doi.org/10.6084/m9.figshare.20073788.v1.

## Ethics Statement

The animal study was reviewed and approved by the Institutional Animal Care and Use Committee of the University of Illinois at Urbana Champaign.

## Author Contributions

HC and GT conceived of the study and participated in its design and coordination. HC, GT, and AW drafted the manuscript. GT and AW carried out the experiments and statistical analyses. JR contributed to the analyses and manuscript preparation. All authors read and approved the final manuscript.

## Funding

This research was supported by the National Institutes of Health under awards R01 NS083402, R01 NS097610, and R01 NS100019 (to HC) and R21 NS104293 and R21 NS109894 (to JR) from the National Institute of Neurological Disorders and Stroke.

## Conflict of Interest

The authors declare that the research was conducted in the absence of any commercial or financial relationships that could be construed as a potential conflict of interest.

## Publisher's Note

All claims expressed in this article are solely those of the authors and do not necessarily represent those of their affiliated organizations, or those of the publisher, the editors and the reviewers. Any product that may be evaluated in this article, or claim that may be made by its manufacturer, is not guaranteed or endorsed by the publisher.
