# Supplementary material for: Heterozygous Deletion of Epilepsy Gene KCNQ2 Has Negligible Effects on Learning and Memory
Source: Front Behav Neurosci. 2022 Jul 19;16:930216. doi: 10.3389/fnbeh.2022.930216 (PMC9344800; doi:10.3389/fnbeh.2022.930216)
Supplement: Supplementary file 1 [file Data_Sheet_1.PDF]

Supplementary Material

Heterozygous deletion of epilepsy gene *KCNQ2* has negligible effects on learning and memory

Gregory C. Tracy[1], Angelina R. Wilton[1], Justin S. Rhodes[2,3,4], Hee Jung Chung[1,2,4*]

[1]Department of Molecular and Integrative Physiology, University of Illinois at Urbana-Champaign, Urbana, IL 61801, USA.

[2]Beckman Institute for Advanced Science and Technology, University of Illinois at Urbana-Champaign, Urbana, IL 61801, USA.

[3]Department of Psychology, University of Illinois at Urbana-Champaign, Urbana, IL 61801, USA.

[4]Neuroscience Program, University of Illinois at Urbana-Champaign, Urbana, IL 61801, USA.

*** Correspondence:**

Hee Jung Chung

Department of Molecular and Integrative Physiology,

University of Illinois at Urbana-Champaign,

407 South Goodwin Avenue, 524 Burrill Hall,

Urbana, IL 61801, USA.

chunghj@illinois.edu

1 Supplementary Figures and Tables

1.1 Supplementary Figures

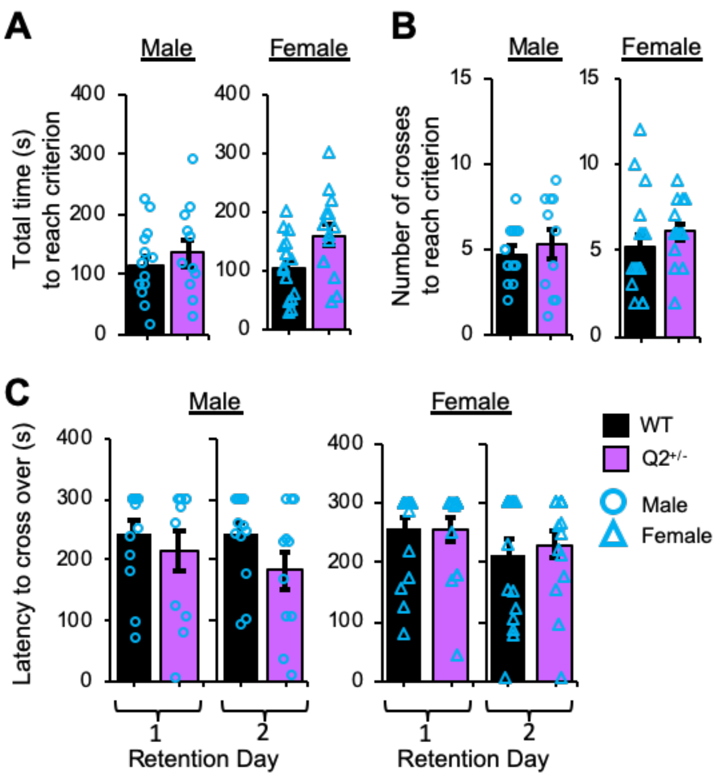

Supplementary Figure 1. Raw data points from the passive inhibitory avoidance test shown in Figure 1. (A) Total time (s) to reach criterion during training. **(B)** Number of crosses to reach criterion during training. **(C)** Latency to cross over at 1 and 2 days after the foot shock. Number of mice used: *KCNQ2*$^{+/+}$ (WT) male (n=14) and female (n=17), *KCNQ2*$^{+/-}$ (Q2$^{+/-}$): male (n=11) and female (n=15). Data represents the mean ± SEM. Open circles and triangles represent individual data points of male and female mice, respectively. Since the 2-way ANOVA test results with genotype as one factor and sex as the other (Table S1) showed no sex differences and no interaction with genotype and sex, Figure 1 shows the analysis of male and female data combined.

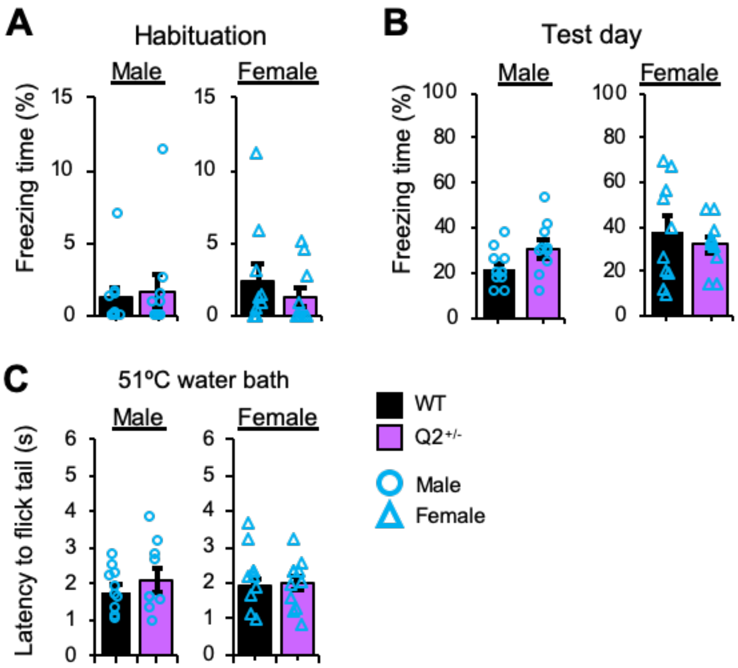

Supplementary Figure 2. Raw data points from the contextual fear conditioning test and tail flick assay shown in Figure 2. (A-B) Percentage (%) of freezing time during habituation and at 1 day after foot shocks in the contextual fear conditioning test. Number of mice used: *KCNQ2*$^{+/+}$ (WT) male (n=14) and female (n=17), *KCNQ2*$^{+/-}$ (Q2$^{+/-}$) male (n=11) and female (n=15). **(C)** Latency to tail flick latency at 51 ºC in tail flick assay. All mice reached the maximum 30 s latency at 36 ºC. Number of mice used: WT male (n=10) and female (n=10), Q2$^{+/-}$ male (n=9) and female (n=11). Data represents the mean ± SEM. Open circles and triangles represent individual data points of male and female mice, respectively. Since the 2-way ANOVA test results with genotype as one factor and sex as the other (Table S1) showed no sex differences and no interaction with genotype and sex, Figure 2 shows the analysis of male and female data combined.

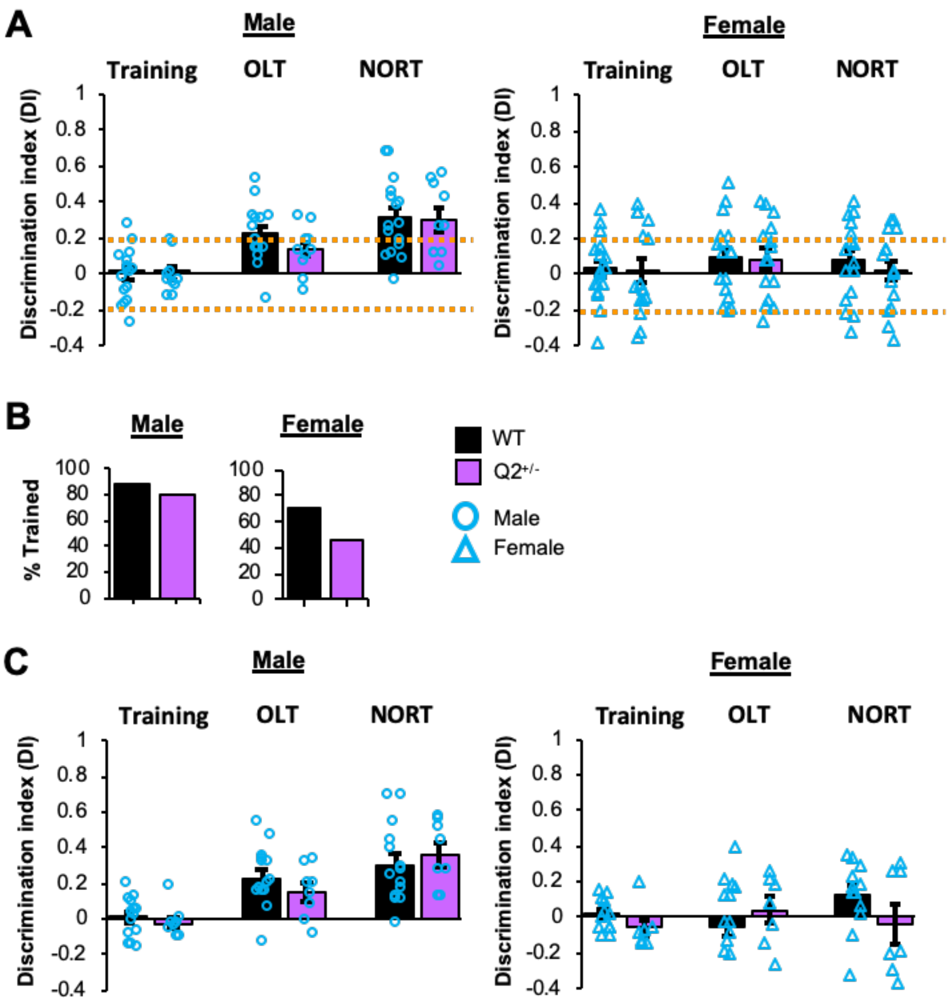

Supplementary Figure 3. Raw data points from the training, object location test, and novel object recognition test shown in Figure 3. (A) All raw data points from the training, object location test (OLT), and novel object recognition test (NORT). The orange dotted lines show the the discrimination index (DI) of + 0.2 (top lines) and - 0.2 (bottom lines). **(B)** Percentage (%) of $KCNQ2^{+/+}$ (WT) and $KCNQ2^{+/-}$ ($Q2^{+/-}$) mice that did not show object bias during training indicated by the Discrimination Index (DI) that were equal to and less than ± 0.2 from the data points shown in (A). Number of mice used in A-B: WT male (n=16) and female (n=17), $Q2^{+/-}$ male (n=10) and female (n=15). **(C)** Data points from the training, OLT, and NORT analyzed in Figure 3 after removing the mice that showed object bias during training (DI > ± 0.2). Open circles and triangles represent individual data points of male and female mice, respectively. Number of mice used: WT male (n=14) and female (n=12). Q2+/- male (n=8) and female (n=7). Data represents the mean ± SEM. Since the 2-way ANOVA test results with genotype as one factor and sex as the other (Supplementary Table 1) showed sex differences and interaction with genotype and sex during OLT and NORT, male and female data were analyzed separately in Figure 3.

1.2 Supplementary Tables

Supplementary Table 1. Two-way ANOVA statistical analysis for Figure S1, S2, and S3C with genotype as one factor and sex as another factor.

Behavior Test	Result	ANOVA table	F(DFn, DFd)	P Value
Passive avoidance	Training	Sex	F(1, 53) = 0.15409	P = 0.69624
	total time to reach criterion (s)	Genotype	F (1, 53) = 4.87579	P = 0.03158
		Interaction	F(1, 53) = 1.01651	P = 0.31793
	Training	Sex	F(1, 55) = 0.01248	P = 0.91144
	Trial-1 Average latency (s)	Genotype	F(1, 55) = 1.88937	P = 0.17485
		Interaction	F(2, 55) = 0.95994	P = 0.38924
	Training	Sex	F(1, 54) = 1.01552	P = 0.31808
	Trial-2 Average latency (s)	Genotype	F(1, 54) = 1.08577	P = 0.30205
		Interaction	F(2, 54) = 0.9831	P = 0.38075
	Training	Sex	F(1,47) = 0.4404	P = 0.51017
	Trial-3 Average latency (s)	Genotype	F(1, 47) = 0.07135	P = 0.79055
		Interaction	F(2, 47) = 0.23609	P = 0.79064
	Training	Sex	F(1, 43) = 0.000700126	P = 0.97901
	Trial-4 Average latency (s)	Genotype	F(1, 43) = 1.24047	P = 0.27157
		Interaction	F(2, 43) = 0.63008	P = 0.53739
	Training	Sex	F(1, 30) = 0.49532	P = 0.48699
	Trial-5 Average latency (s)	Genotype	F(1, 30) = 4.7831	P = 0.03667
		Interaction	F(2, 30) = 2.47578	P = 0.10112
	Training	Sex	F(1, 26) = 0.00496	P = 0.94439
	Trial-6 Average latency (s)	Genotype	F(1, 26) = 2.33815	P = 0.13832
		Interaction	F(2, 26) = 1.16948	P = 0.32633
	Training	Sex	F(1, 13) = 0.18925	P = 0.67068
	Trial-7 Average latency (s)	Genotype	F(1,13) = 0.35138	P = 0.5635
		Interaction	F(2, 13) = 0.32074	P = 0.73119
	Training	Sex	F(1, 10) = 0.18536	P = 0.67594
	Trial-8 Average latency (s)	Genotype	F(1, 10) = 0.03472	P = 0.85592
		Interaction	F(2, 10) = 0.119	P = 0.88905
	Training	Sex	F(1, 2) = 0.10559	P = 0.77606
	Trial-9 Average latency (s)	Genotype	F(1,2) = 0.26389	P = 0.65859
		Interaction	F(2, 2) = 0.13203	P = 0.88337
	Training	Sex	F(1, 53) = 1.06474	P = 0.30682
	number of crosses (n)	Genotype	F(1, 53) = 1.18943	P = 0.28038
		Interaction	F(1, 53) = 0.04587	P = 0.83123
	Latency to cross over (s)	Sex	F(1, 53) = 1.25263	P = 0.2681
	Day 1	Genotype	F(1, 53) = 0.48711	P = 0.48827
		Interaction	F(1, 53) = 0.42756	P = 0.51601
	Latency to cross over (s)	Sex	F(1, 53) = 0.09966	P = 0.75348

	Day 2	Genotype	F(1, 53) = 0.79593	P = 0.37635
		Interaction	F(1, 53) = 2.34891	P = 0.42097
Contextual fear conditioning	Freezing time (%) before training	Sex	F(1, 35) = 2.07153	P = 0.15896
		Genotype	F(2,35) = 1.08102	P = 0.35031
		Interaction	F(2, 35) = 1.47768	P = 0.24205
	Freezing time (%) after training	Sex	F(1, 36) = 3.15391	P = 0.0842
		Genotype	F(1, 36) = 0.16988	P = 0.68267
		Interaction	F(1, 36) = 2.23956	P = 0.14324
Tail flick 51C	Latency to flick tail (s)	Sex	F(1, 36) = 0.10205	P = 0.75123
		Genotype	F(1, 36) = 0.06318	P = 0.80298
		Interaction	F(1, 36) = 1.06394	P = 0.3092
T, OLT, NORT Mice without object bias during training	Training	Sex	F(1, 38) = 0.00222	P = 0.96269
		Genotype	F(1, 38) = 2.14734	P = 0.15104
		Interaction	F(2, 38) = 1.07515	P = 0.3514
	OLT	Sex	F(1, 38) = 7.90342	P = 0.00776
		Genotype	F(1, 38) = 0.80859	P = 0.3742
		Interaction	F(2, 38) = 4.36864	P = 0.0196
	NORT	Sex	F(1, 38) = 12.99823	P = 8.92989E-4
		Genotype	F(1, 38) = 0.39497	P = 0.53346
		Interaction	F(2, 38) = 6.70799	P = 0.0032

Supplementary Table 2. Two-way ANOVA statistical analysis for Figure S3C with genotype as one factor and task as another factor.

Behavior Test	Result	ANOVA table	F(DFn, DFd)	P Value
T, OLT, NORT All males	DI	Genotype	F(1, 72) = 0.82482	P = 0.3668
		Task	F(2, 72) = 21.09827	P = 6.144E-8
		Interaction	F(2, 72) = 0.49872	P = 5.643E-7
T, OLT, NORT All females	DI	Genotype	F(1, 91) = 0.34023	P = 0.56114
		Task	F(2, 91) = 0.71366	P = 0.49257
		Interaction	F(2, 91) = 0.13969	P = 0.84257
T, OLT, NORT Males without object bias during training	DI	Genotype	F(1, 62) = 0.23351	P = 0.63063
		Task	F(2,62) = 22.48828	P = 4.531E-8
		Interaction	F(3, 62) = 15.07002	P = 1.785E-7
T, OLT, NORT Females without object bias during training	DI	Genotype	F(1, 53) = 2.61756	P = 0.11162
		Task	F(2, 53) = 0.90022	P = 0.4126
		Interaction	F(3, 53) = 1.47267	P = 0.23249
